# Conference Calendar

**DOI:** 10.1002/cfg.489

**Published:** 2005


					Comparative and Functional Genomics
Comp Funct Genom 2005; 6: 323-325.
Published online in Wiley InterScience (www.interscience.wiley.com). DOI: 10.1002/cfg.489
Conference Calendar
January-March 2006
Animal Models
GSA -- Genetic Analysis: Model Organisms to
Human Biology, January 5-7
http://www.gsa-modelorganisms.org/
Plant and Animal Genome XIV Conference,
January 14-18
http://www.intl-pag.org
47th Annual Drosophila Research Conference,
March 29-April 2
http://genetics.faseb.org/genetics/g-gsa/
future meetings.shtml
Bioinformatics
Pacific Symposium on Biocomputing, January
3-7
http://psb.stanford.edu/
5th Annual Hawaii International Conference on
Statistics, Mathematics and Related Fields,
January 16-18
http://www.hicstatistics.org
Biocomputation and Physics of Complex
Systems (BIFI) 2006, February 8-11
http://bifi.unizar.es/events/bifi2006/index.html
4th Asia-Pacific Bioinformatics Conference,
February 13-16
http://binfo.ym.edu.tw/apbc2006/
Functional Genomics
KEY -- RNAi and Related Pathways, January
26-31
http://www.keystonesymposia.org/Meetings/
ViewMeetings.cfm?MeetingID=813
Human Genomics and Genetics
KEY -- Genome sequence variation and the
inherited basis of common disease and complex
traits, January 8-13
http://www.keystonesymposia.org/Meetings/
ViewMeetings.cfm?MeetingID=787
General
Society for Integrative and Comparative
Biology Annual Meeting, January 4-8
http://www.sicb.org/meetings/2006/
index.php3
GRC -- Reversible Associations in Structural &
Molecular Biology, January 15-20
http://www.grc.uri.edu/programs/2006/
revers.htm
GRC -- Molecular Evolution, February 5-10
http://www.grc.uri.edu/06sched.htm
GRC -- Genes & Behavior, February 12-17
http://www.grc.uri.edu/06sched.htm
ASM -- Conference on Mobile DNA,
February 24-March 2
http://www.asm.org/Meetings/
Metabolomics
1st Maga Circe Conference on Metabolic
Systems Analysis, March 26-29
http://www.chem.uniroma1.it/magacirce
conferences/
Copyright  2005 John Wiley & Sons, Ltd.
324 Conference Calendar
Microbes
KEY -- Microbial Community Genomics in
Animals and in the Environment,
February 6-10
http://www.keystonesymposia.org/Meetings/
ViewMeetings.cfm?MeetingID=788
KEY -- Malaria: Functional Genomics to
Biology to Medicine, February 28-March 5
http://www.keystonesymposia.org/Meetings/
ViewMeetings.cfm?MeetingID=804
ASM -- 8th Conference on Candida and
Candidiasis, March 13-17
http://www.asm.org/Meetings/
ASM -- Conference on Dimorphic Fungal
Pathogens, March 13-17
http://www.asm.org/Meetings/
WT -- European Fission Yeast Meeting,
March 16-18
http://www.wellcome.ac.uk/doc wtx024117.html
Pharmacogenomics/Genomics and
Disease
KEY -- The Impact of Chemogenomics on
Drug Discovery and Development,
January 23-27
http://www.keystonesymposia.org/Meetings/
ViewMeetings.cfm?MeetingID=772
CHI -- Molecular Medicine Tri-Conference,
February 21-24
http://www.chimolecularmed.com/
GRC -- New Antibacterial Discovery &
Development, March 5-10
http://www.grc.uri.edu/programs/2006/
antibact.htm
Plants
Plant and Animal Genome XIV Conference,
January 14-18
http://www.intl-pag.org
Proteomics
CHI -- PepTalk: The Plasma Proteome;
Protein Expression; Advancing Protein
Therapeutics; Protein Arrays; Protein Process
Development, January 9-13
http://www.chi-peptalk.com/
GRC -- Peptides, Chemistry & Biology Of,
February 19-24
http://www.grc.uri.edu/programs/2006/
peptides.htm
57th Pittsburgh Conference on Analytical
Chemistry and Applied Spectroscopy
(PITTCON), March 12-17
http://www.pittcon.org/
Structural Genomics
KEY -- Structural Genomics, January
29-February 3
http://www.keystonesymposia.org/Meetings/
ViewMeetings.cfm?MeetingID=817
KEY -- Frontiers in Structural Biology, January
29-February 3
http://www.keystonesymposia.org/Meetings/
ViewMeetings.cfm?MeetingID=816
Systems Biology
KEY -- Systems Biology: Integrating Biology,
Technology and Computation, March 5-10
http://www.keystonesymposia.org/Meetings/
ViewMeetings.cfm?MeetingID=789
Copyright  2005 John Wiley & Sons, Ltd. Comp Funct Genom 2005; 6: 323-325.
Conference Calendar 325
CSBMCB -- 48th Annual Meeting on Cellular
Dynamics, March 16-20
http://www.csbmcb.ca/2004Meeting/index.html
Biotech Industry
Select Conferences -- Screening Europe &
Medchem Europe, February 20-22
http://www.selectbiosciences.com/conferences/
screeningeurope/
Key:
ASM: American Society for Microbiology: http://
www.asm.org/
CHI: Cambridge Healthtech Institute: http://www.
healthtech.com
CSBMCB: Canadian Society for Biochemistry
and Molecular & Cellular Biology: http://www.
csbmcb.ca/
GRC: Gordon Research Conferences: http://www.
grc.uri.edu/
GSA: Genetics Society of America: http://www.
genetics-gsa.org/
KEY: Keystone Symposia: http://www.keystone
symposia.org/
WT: Wellcome Trust: http://www.wellcome.
ac.uk/
The Conference Calendar is a listing of forthcoming conferences of interest to our readership. We provide
the title, dates and web address of each conference, to enable readers to find out more information.
Inclusion does not imply endorsement by the journal. If you know of a relevant conference, please
contact the Managing Editor.
Copyright  2005 John Wiley & Sons, Ltd. Comp Funct Genom 2005; 6: 323-325.

				

